# How nasopharyngeal pneumococcal carriage evolved during and after a PCV13-to-PCV10 vaccination programme switch in Belgium, 2016 to 2018

**DOI:** 10.2807/1560-7917.ES.2020.25.5.1900303

**Published:** 2020-02-06

**Authors:** Ine Wouters, Stefanie Desmet, Liesbet Van Heirstraeten, Sereina A Herzog, Philippe Beutels, Jan Verhaegen, Herman Goossens, Pierre Van Damme, Surbhi Malhotra-Kumar, Heidi Theeten, NPcarriage Study Group

**Affiliations:** 1 Centre for the Evaluation of Vaccination, Vaccine and Infectious Disease Institute, University of Antwerp, Wilrijk, Belgium; 2 Reference Centre for Pneumococci, University Hospitals Leuven, Leuven, Belgium; 3 Laboratory of Medical Microbiology, Vaccine and Infectious Disease Institute, University of Antwerp, Wilrijk, Belgium; 4 Centre for Health Economics Research and Modelling Infectious Diseases, University of Antwerp, Wilrijk, Belgium; 5 The members of the NPcarriage Study Group are listed at the end of the article

**Keywords:** Nasopharyngeal carriage, *Streptococcus pneumoniae*, *Haemophilus influenzae*, Pneumococcal conjugate vaccines, Children, Day-care centres

## Abstract

**Background:**

The current carriage study was set up to reinforce surveillance during/after the PCV13-to-PCVC10 switch in Belgium.

**Aim:**

This observational study monitored carriage of *Streptococcus pneumoniae* (Sp) serotypes, particularly those no longer covered (3, 6A, 19A), as well as *Haemophilus influenzae* (Hi), because PCV10 contains the non-typeable Hi protein D.

**Methods:**

A total of 2,615 nasopharyngeal swabs from children (6–30 months old) attending day care were collected in three periods over 2016–2018. Children’s demographic and clinical characteristics and vaccination status were obtained through a questionnaire. Sp and Hi were identified by culture and PCR. Pneumococcal strains were tested for antimicrobial (non-)susceptibility by disc diffusion and serotyped by Quellung-reaction (Quellung-reaction and PCR for serotypes 3, 6A, 19A).

**Results:**

The carriage prevalence of Sp (> 75%) remained stable over the successive periods but that of Hi increased (87.4%, 664 Hi-carriers/760 in 2016 vs 93.9%, 895/953 in 2017–2018). The proportion of non-PCV13 vaccine serotypes decreased (94.6%, 438 isolates/463 in 2016 vs 89.7%, 599/668 in 2017–2018) while that of PCV13-non-PCV10 vaccine serotypes (3 + 6A + 19A) increased (0.9%, 4 isolates/463 in 2016 vs 7.8%, 52/668 in 2017–2018), with serotype 19A most frequently identified (87.9%, 58/66 isolates). Non-susceptibility of pneumococci against any of the tested antibiotics was stable over the study period (> 44%).

**Conclusions:**

During and after the PCV13-to-PCV10 vaccine switch, the proportion of non-PCV13 serotypes decreased, mainly due to a serotype 19A carriage prevalence increase. These results complement invasive pneumococcal disease surveillance data, providing further basis for pneumococcal vaccination programme policy making.

## Introduction

Nasopharyngeal carriage of *Streptococcus pneumoniae* (Sp) frequently occurs asymptomatically [1-5]. Nevertheless, it may evolve to respiratory infections such as otitis media and pneumonia or even invasive diseases including bacteraemia and meningitis [2,3,5]. Besides the elderly, young children are prone to (invasive) pneumococcal diseases ((I)PD) [6-10]. Before pneumococcal conjugate vaccines (PCVs) were introduced, the global annual number of serious pneumococcal disease cases (pneumonia, meningitis, and bacteraemia) in children under 5 years of age was estimated to be 14.5 million [11].

The primary virulence factor of Sp is its polysaccharide capsule, which also determines the serotype. More than 95 serotypes exist and they vary in their capacity to activate the host immune system and to invade [12-15]. PCVs provide direct protection to the vaccinated individuals against a number of clinically relevant serotypes [12]. In addition, the wider population experiences indirect protection against pneumococcal disease through reduced nasopharyngeal carriage of pneumococcal vaccine serotypes (VTs). However, the observed magnitude of this indirect effect varies in different contexts, and it is eroded by the rising incidence of non-VT-(NVT-)related diseases [16]. Several studies on carriage or IPD in the pre- and post-PCV era reported on serotype replacement, i.e. VTs being largely replaced by NVTs [17,18]. Furthermore, co-colonisation with other pathogens such as *Haemophilus influenzae* (Hi), *Moraxella catarrhalis* (Mc), *Staphylococcus aureus* (Sa), and *Streptococcus pyogenes* (GAS) may be changed after PCV-introduction because of mutual interactions [19-21].

Belgium initiated a universal childhood PCV-programme according to a two plus one schedule in 2007 (at 8 weeks, 16 weeks, and 12 months of age). The seven-valent vaccine (PCV7, including serotypes 4, 6B, 9V, 14, 18C, 19F, 23F) was superseded by the 13-valent vaccine (PCV13, including PCV7 serotypes plus 1, 5, 7F, 3, 6A, 19A, same 2 + 1 schedule) in 2011, which was in turn replaced by the 10-valent vaccine (PCV10, including PCV7 serotypes plus 1, 5, 7F, same 2 + 1 schedule) in 2015–2016. The implementation of immunisation programmes constitutes a regional responsibility in Belgium. PCV10 was introduced in the Flemish (Northern) region in July 2015 and in the Walloon (Southern) region in May 2016 [22]. In the Brussels (Capital) region either the Flemish or the Walloon programme was followed, depending on the consulting physician. The pneumococcal vaccination programme rapidly achieved high three-dose coverage in children (coverage in Belgium; > 80% in all regions in 2008–2009 vs > 94% in all regions in 2015–2016 [23-26]) and the overall incidence of IPD in Belgium significantly decreased after implementation of the vaccination programme; post-PCV7 period (2007–2010) vs pre-PCV7 period (pre 2007): decrease of 35%; post-PCV13 (2015) vs PCV7-era (2007–2010): decrease of 42% [22].

The current carriage study was set up to reinforce surveillance after the PCV13-to-PCV10 vaccination programme switch, in order to monitor the three pneumococcal serotypes that were no longer covered (3, 6A, 19A), as well as Hi, because PCV10 contains the non-typeable Hi (NTHi) protein D. To this end, we studied nasopharyngeal carriage of Sp and Hi in children between 6 and 30 months of age attending day care centres (DCCs) during three consecutive periods between 2016 and 2018. High pneumococcal carriage rates (range: 21–89%) have been reported in young children attending day care [17,27-29]. As such, the impact of the PCV-programme change was monitored in a random sample of this target population, to complement sentinel laboratory-reported IPD-surveillance. In this paper, we focus on pneumococcal serotype distribution and antimicrobial (non-)susceptibility during and after the PCV13-to-PCV10 vaccination programme switch.

## Methods

### Ethical statement

The current study was in line with the Declaration of Helsinki, as revised in 2013. Approval to conduct the current study with ID 15/45/471 was obtained from the University of Antwerp and University Hospital of Antwerp ethics committee (Commissie voor Medische Ethiek van UZA/UA) on 30 November 2015.

### Study design

The design of this observational study was previously described in detail and is summarised here for the complete study period (from Period 1 in 2016 up to Period 3 in 2017–2018) [30,31].

Nasopharyngeal sampling was performed between March and July in Period 1 (2016) and between November and March in the consecutive periods (Period 2: 2016–2017 and Period 3: 2017–2018). Healthy children were recruited in DCCs randomly selected over the three Belgian regions (Wallonia, Flanders, Brussels), according to a population-proportionate distribution at regional level based on Belgian Federal Government Statistics for the 0–4 year population. In the consecutive periods, 85, 112, and 102 DCCs participated in the study, of which 66 DCCs participated in all three periods, 44 in two periods, and 24 in one period. A population-proportionate sample at the regional level was achieved from 2016 to 2017 onwards, after deliberate over-recruitment in Wallonia in 2016, in order to include a maximum of children who received PCV13 for both primary vaccine doses and their booster, since at that time, PCV10 was not yet introduced in Wallonia. The inclusion criteria were: no treatment with oral antibiotics (ABs) in the 7 days before sampling and age between 6 and 30 months included. An additional age criterion (date of birth before 1 January 2015) was applied in Flanders and Brussels for 2016, in order to exclusively include children who received PCV13 for their primary vaccine doses [30]. In this way, children recruited in the first period were on average older compared with children recruited in the subsequent periods.

Trained nurses and physicians conducted a questionnaire collecting demographic and clinical characteristics of the study participants. The vaccination status of the participating child was based on vaccination documentation or parental reporting. A single nasopharyngeal swab was taken with a flocked nylon fibre swab, transported in 1 mL skim milk-tryptone-glucose-glycerol (STGG) and cultured or stored at − 80 °C within 24 hours.

### Culture analyses

At the National Reference Centre (NRC) for pneumococci, nasopharyngeal samples were plated on blood agar plates for identification of Sp, Mc, Sa, GAS and a selective plate for identification of Hi, following overnight enrichment in brain-heart infusion (BHI) broth (entire study period for blood agar plates, 2016 only for selective plate) and directly (from 2016–2017 onwards for blood agar plates and selective plate). Sp*-*strains were serotyped via Quellung-reaction. Antimicrobial (non-)susceptibility of the Sp-strains for erythromycin, levofloxacin, penicillin, tetracycline and trimethoprim/sulfamethoxazole was determined by disc diffusion according to the guidelines of Clinical and Laboratory Standards Institute (CLSI; 2016 and 2016–2017) [32] and European Committee on Antimicrobial Susceptibility Testing (EUCAST; 2017–2018) [33]. If non-susceptibility for penicillin or levofloxacin was identified by disc diffusion, the minimum inhibitory concentration (MIC) was determined by Etest (Biomérieux, Craponne, France). A MIC of > 0.06 mg/L for penicillin or > 2 mg/L for levofloxacin was interpreted as non-susceptible.

### Molecular analyses

DNA was extracted from 200 µL of nasopharyngeal sample and tested in real-time PCR targeting *lytA* (for Sp) or *P6* (for Hi) [30,34,35]. Real-time PCR was performed for Sp on all samples and for Hi on culture-negative samples. Samples were classified as positive for Sp or Hi when cycle threshold (C_T_) values were ≤ 40 or ≤ 35, respectively. *LytA*-positive samples were pooled and screened for presence of the three pneumococcal serotypes included in PCV13, but not in PCV10 (PCV13-non-PCV10-VTs: 3, 6A, 19A). If found positive for serotype 3, 6A, or 19A, pooled samples were unpooled and positivity of the individual sample was determined. Serotype-specific PCRs were performed in a 20 µL reaction volume containing 2x Taqman Universal PCR Master Mix (Applied Biosystems), 200 nM concentrations of serotype 3 [36], 6A [37], or 19A [36] primers and probe and 2 µL of DNA template (pooled PCR-reaction contains four times 2 µL of DNA). Samples positive for 6A real-time PCR were further subjected to 6C real-time PCR targeting *wciN*_β_ to discriminate between serotypes 6A and 6C [38]. The serotype-specific 6C assay was performed as described above, with the exception that higher primer concentrations of 500 nM were used. Amplification was carried out on a StepOnePlus Real-Time PCR System (Applied Biosystems, Foster City, California, United States) using the following cycling parameters: 10 min at 95 °C and 40 cycles of 15 s at 95 °C and 1 min at 59.5 °C (3, 19A) or 60 °C (6A, 6C). Serotype-specific PCRs were classified as positive when C_T_ values were ≤ 35.

Samples were considered positive for any of the respective pathogens if either culture or PCR was positive. The presented carriage prevalences of Sp and Hi were based on culture and PCR-results, whereas overall serotype distribution and antimicrobial (non-)susceptibility were based on Quellung-reaction and culture results respectively. The reported carriage prevalence of serotypes 3, 6A, and 19A was based on Quellung-reaction and PCR-results.

### Statistics

Sample size and power were calculated using the R-package ‘power’ [30]. A sample size of 700 children in 2016 and 900 children from the second period onwards allows the detection of 4% changes in carriage prevalence of Sp-serotypes 19A or 6A over the observation period of the study period with 80% power and assuming a starting carriage prevalence below 2%.

In IBM SPSS Statistics 25, the chi-squared (Chi^2^) or Fisher’s Exact Test (FET) and the Mann–Whitney U Test (MWU) were used to test for significance at a level of 5%. To identify predictors of carrying Sp, PCV13-non-PCV10-VTs, and Hi in DCC-children (three periods pooled), univariate and multiple binary logistic regressions were performed and adjusted using generalised estimating equations (GEEs) with an exchangeable correlation structure since 148 children (303 isolates) contributed more than one sample over the 3-year study period. The GEE model analyses were performed using the statistical software R (version 3.6.1) with the geepack package (version 1.2–1). Variables with a p value < 0.1 in the univariate analysis were included in the multiple regression analysis. Since no children were sampled in the youngest age category in Flanders and in Brussels in 2016, no adjustments can be made for the different sampling probabilities in the different study years. A continuity correction was applied for 95% confidence intervals (95% CI) on proportions. Missing values were not replaced.

## Results

### Study population

Over the three successive periods, nasopharyngeal samples from 2,883 children attending DCCs were collected. In total, 2,615 samples (760 in 2016, 902 in 2016–2017, 953 in 2017–2018) – corresponding to 2,621 pneumococcal isolates, as more than one serotype could be found per child (761 in 2016, 904 in 2016–2017, 956 in 2017–2018), were included in the final analyses, i.e. after exclusion of a random selection of 194 samples collected in 2016–2017 (not analysed by PCR as a consequence of over-recruitment), and after exclusion of 74 samples not fulfilling the inclusion criteria regarding age or use of ABs. Of the 2,615 nasopharyngeal samples, 148 (5.7%) originated from children who contributed more than one sample, but never in the same period. The univariate and multiple binary logistic regression models were adjusted for this through GEEs with an exchangeable correlation structure.

The main demographic and clinical characteristics of the child population over the study period are shown in Table 1, with the majority of these characteristics remaining similar over the study period. Nevertheless, a decreasing trend (p < 0.001) was observed for history of acute otitis media (AOM-history) and AB-use in the 3 months before sampling. The proportions of children being breastfed for more than 6 months (p = 0.026) and with symptoms (runny nose and/or cough) of common cold (p < 0.001) increased over the study period. 

**Table 1:** Demographic and clinical characteristics of the healthy child population in day care per period, Belgium, 2016–2018 (n = 760 children in 2016, 902 in 2016–2017, 953 in 2017–2018)

Characteristics		Healthy children attending day care						
		Period 1 2016 (N = 760)		Period 2 2016–2017 (N = 902)		Period 3 2017–2018 (N = 953)		p value chi^2^ for trend
		n	%^a^	n	%^a^	n	%^a^	
Region	Wallonia	353	46.4	287	31.8	282	29.6	**< 0.001**
	Flanders	332	43.7	488	54.1	552	57.9	
	Brussels	75	9.9	127	14.1	119	12.5	
Age in months	6–12	98	12.9	217	24.1	209	21.9	**< 0.001**
	13–24	415	54.6	457	50.7	528	55.4	
	25–30	247	32.5	228	25.3	216	22.7	
Sex	Male	387	50.9	455	50.4	469	49.2	0.474
Preterm delivery	Yes	60	8.0	71	7.9	78	8.2	0.872
Breastfeeding^b^	Yes	230	30.4	289	32.1	336	35.4	**0.026**
Parental smoking^c^	Yes	170	22.4	183	20.4	190	20.0	0.231
Siblings	Yes	459	62.9	548	61.0	599	63.3	0.813
Common cold symptoms	Yes	169	22.4	344	38.2	429	45.0	**< 0.001**
AOM-history^d^	Yes	258	34.8	225	25.5	199	21.8	**< 0.001**
AB < 3 months^e^	Yes	248	35.4	254	30.5	217	23.5	**< 0.001**

AB: antibiotic; AOM: acute otitis media.

^a^ Due to missing information on some characteristics for some children, the denominator can at times differ from ‘N’ in the column heading.

^b^ Child was breastfed for more than 6 months.

^c^ At least one parent smokes.

^d^ Child with a history of AOM based on parental recall.

^e^ Use of antibiotics in the 3 months before sampling.

Significant p values (<0.05) are indicated in bold.

**Figure 1:**
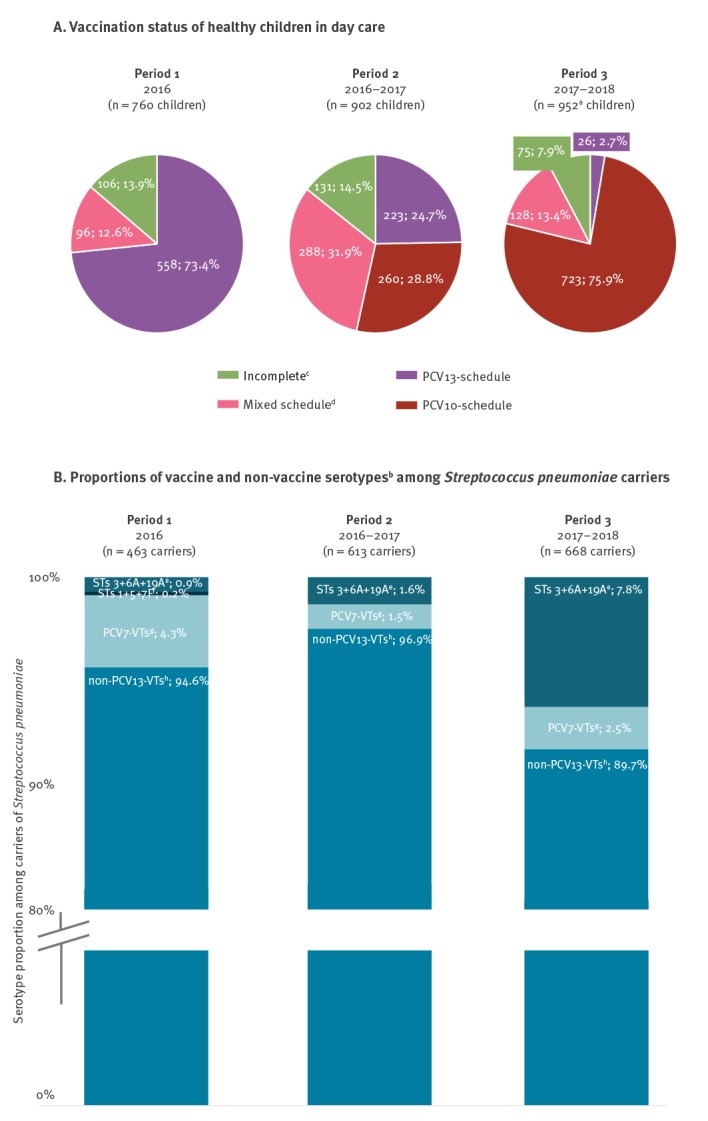
(A) Vaccination status of healthy children in day care (n = 760 in 2016, 902 in 2016–2017, 952^a^ in 2017–2018) and (B) proportions of vaccine and non-vaccine serotypes^b^ among *Streptococcus pneumoniae* carriers (n = 463 carriers in 2016, 613 in 2016–2017, 668 in 2017–2018), Belgium, 2016–2018

As a result of the recruitment strategy to include older children in 2016 (mean age: 21.0 months in 2016 vs 18.4 months in 2016–2017 vs 18.4 months in 2017–2018), the majority of the Period 1 population was vaccinated with PCV13 only (Figure 1: 73.4%; 558/760 children). The proportion of PCV13-vaccinated children decreased over the study period to 2.7% (26/952 children; vaccination status was missing for one of the 953 children in 2017–2018), whereas the proportion of PCV10-vaccinated children increased from 0.0% to 75.9% (723/952 children).

PCV7/10/13: 7/10/13-valent pneumococcal conjugate vaccine; STs: serotypes; VTs: vaccine serotypes.

^a^ A total of 952 is used because vaccination status was missing for one of the 953 children in 2017–2018.

^b^ Serotypes are determined by Quellung reaction.

^c^ Incomplete schedule: children who were not or incompletely vaccinated.

^d^ Mixed schedule: children vaccinated with a combination of PCV13 and PCV10.

^e ^STs 3 + 6A + 19A: serotypes included in PCV13, but not in PCV10.

^f^ STs 1 + 5 + 7F: serotypes included in PCV10 but not in PCV7.

^g^ PCV7-VTs: vaccine serotypes included in PCV7 (4, 6B, 9V, 14, 18C, 19F, 23F).

^h ^Non-PCV13-VTs: vaccine serotypes not included in PCV13.

Children in the study were 6–30 months old. Vaccination status was based on vaccination documentation or parental reporting.

### Carriage prevalence of *Streptococcus pneumoniae* and *Haemophilus influenzae*

**Figure 2:**
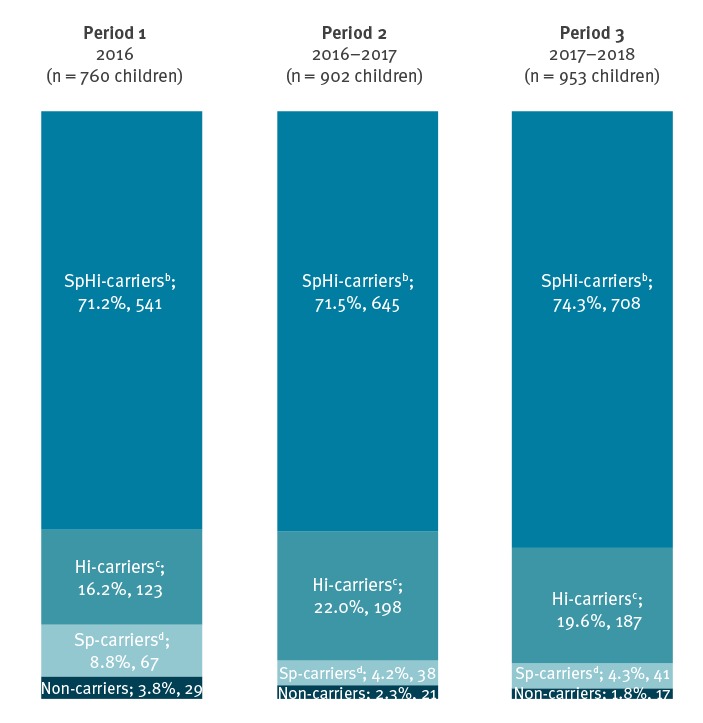
Carriage prevalence^a^ of *Streptococcus pneumoniae* and *Haemophilus influenzae* among healthy children in day care, Belgium, 2016–2018 (n = 760 children in 2016, 902 in 2016–2017, 953 in 2017–2018)

As determined by PCR, very few children carried neither Sp, nor Hi, namely 3.8% (29/760 children), 2.3% (21/902 children), 1.8% (17/953 children) in the consecutive periods (Figure 2). The carriage prevalence of Sp was stable and high, ranging from 75.7% Sp-carriers (683/902 children) in 2016–2017 to 80.0% Sp-carriers (608/760 children) in 2016. The carriage prevalence of Hi increased significantly (p < 0.001) over the study period (87.4% Hi-carriers, 664/760 in 2016 vs 93.5% Hi-carriers, 843/902 in 2016–2017 vs 93.9% Hi-carriers, 895/953, in 2017–2018). Co-colonisation with Sp and Hi was frequent and did not change over the study period; it ranged from 71.2% (541/760 Sp and Hi-carriers) in 2016 to 74.3% (708/953 Sp and Hi-carriers) in 2017–2018.

Hi: *Haemophilus influenza*; Sp: *Streptococcus pneumoniae*.

^a^ Prevalence was inferred from results of culture and PCR combined.

^b ^SpHi-carriers: carriers of Sp and Hi.

^c ^Hi-carriers: carriers of Hi.

^d ^Sp-carriers: carriers of Sp.

In a multiple regression analysis using pooled data of the three periods, positive predictors for Sp-carriage among DCC-children (Table 2) were being sampled in 2016, being female, having symptoms of common cold, carrying Hi or Mc, and having siblings, whereas AB-use in the 3 months before sampling and carrying Sa were associated with a lower likelihood of Sp-carriage. Predictors of carrying PCV13-non-PCV10-VTs (based on Quellung-reaction) were similarly evaluated using binary logistic regression. The only variable that remained significant in the multiple regression model (Table 2) was study period, indicating higher PCV13-non-PCV10-VT-carriage in 2017–2018 compared with the previous periods. The predictors for Hi-carriage that were significant in the multiple regression model were having symptoms of common cold (odds ratio (OR): 1.63; 95% CI: 1.15–2.31), having siblings (OR: 1.45; 95% CI: 1.08–1.94), and carrying Sp (OR: 1.54; 95% CI: 1.12–2.12), Mc (OR: 1.70; 95% CI: 1.11–2.59), or GAS (OR: 8.52; 95% CI: 1.17–62.00) and all of these were positive predictors. Being in the age category of 13–24 months (compared to the two other age categories: 6–12 months (OR: 0.42; 95% CI: 0.29–0.60) and 25–30 months (OR: 0.59; 95% CI: 0.42–0.83)), or being sampled in 2016–2017 (OR: 2.28; 95% CI: 1.60–3.26) or 2017–2018 (OR: 2.26; 95% CI: 1.58–3.24, compared with 2016) was associated with a higher likelihood of Hi-carriage. Study period and vaccination schedule were both included in all regression analyses since no collinearity between these variables was observed based on a linear regression analysis.

**Table 2:** Predictors through binary logistic regression of *Streptococcus pneumoniae* carriage^a^ (n = 2,615 nasopharyngeal samples) and of PCV13-non-PCV10 vaccine serotype carriage^b^ (n = 1,744) among children attending day care (pooled over study periods), Belgium, 2016–2018

Number of samples/isolates^c^			Univariate regression			Multiple regression		
Characteristic	n	%	OR	95% CI^d^		OR		95% CI^d^
Predictors of Sp-carriage^a^								
Study period								
2016	760	29.1	REF		REF		REF	REF
2016–2017	902	34.5	**0.78**		**0.62–0.99**		**0.64**	**0.47–0.87**
2017–2018	953	36.4	0.92		0.73–1.16		**0.71**	**0.52–0.96**
Region								
Wallonia	922	35.3	REF		REF		REF	REF
Flanders	1,372	52.5	**1.29**		**1.06–1.57**		1.09	0.83–1.42
Brussels	321	12.3	**1.42**		**1.04–1.95**		1.16	0.81–1.66
Sex								
Female	1,304	49.9	REF		REF		REF	REF
Male	1,311	50.1	**0.75**		**0.63–0.91**		**0.76**	**0.62–0.93**
Common cold symptoms^e^								
Yes	942	36.1	REF		REF		REF	REF
No	1,668	63.9	**0.65**		**0.53–0.80**		**0.64**	**0.51–0.80**
Sa-carriage^f^								
Yes	116	4.4	REF		REF		REF	REF
No	2,499	95.6	**1.71**		**1.14–2.55**		**1.79**	**1.13–2.85**
Hi-carriage^g^								
Yes	2,402	91.9	REF		REF		REF	REF
No	213	8.1	**0.58**		**0.43–0.79**		**0.64**	**0.45–0.90**
Mc-carriage^g^								
Yes	2,382	91.1	REF		REF		REF	REF
No	233	8.9	**0.26**		**0.19–0.34**		**0.31**	**0.23–0.42**
Siblings^h^								
Yes	1,606	62.4	REF		REF		REF	REF
No	969	37.6	**0.72**		**0.59–0.87**		**0.73**	**0.61–0.89**
AOM-history^I,j^								
Yes	682	26.9	REF		REF		REF	REF
No	1,855	73.1	**1.36**		**1.11–1.67**		1.14	0.91–1.44
AB-use < 3 months^k,l^								
Yes	719	29.3	REF		REF		REF	REF
No	1,739	70.7	**1.79**		**1.47–2.18**		**1.63**	**1.30–2.05**
Age (months)								
6–12	524	20.0	0.81		0.64–1.03		0.94	0.73–1.22
13–24	1,400	53.5	REF		REF		REF	REF
25–30	691	26.4	**0.79**		**0.64–0.99**		0.84	0.65–1.07
Sampled during influenza-peak								
Yes	1,072	41.0	REF		REF		REF	REF
No	1,543	59.0	**1.23**		**1.02–1.48**		1.01	0.77–1.33
Predictors of PCV13-non-PCV10-VT-carriage^b^								
Study period								
2016	463	26.5	REF		REF		REF	REF
2016–2017	613	35.1	1.90		0.59–6.11		1.36	0.36–5.07
2017–2018	668	38.3	**9.69**		**3.48–27.00**		**5.88**	**1.56–22.19**
Vaccination schedule^m^								
PCV13	486	27.9	REF		REF		REF	REF
PCV10	707	40.6	**6.85**		**2.70–17.35**		1.79	0.53–6.01
Incomplete	222	12.7	1.77		0.47–6.64		0.92	0.22–3.87
Mix	328	18.8	**3.03**		**1.02–8.93**		1.71	0.50–5.82
Sampled during RSV-peak								
Yes	601	34.5	REF		REF		REF	REF
No	1143	65.5	**0.512**		**0.313-0.838**		0.88	0.52–1.50

AB: antibiotic; AOM: acute otitis media; CI: confidence interval; Hi: *Haemophilus influenza*; Mc: *Moraxella catarrhalis*; OR: odds ratio; PCV: pneumococcal conjugate vaccine; PCV13-non-PCV10-VT: vaccine serotypes included in PCV13, but not in PCV10 (serotypes: 3, 6A, 19A); REF: reference group for the regression analysis; RSV: respiratory syncytial virus; Sa: *Staphylococcus aureus*; Sp: *Streptococcus* *pneumoniae*.

^a^ Knowledge of Sp carriage was through results of culture and PCR combined.

^b^ Knowledge of PCV13-non-PCV10 vaccine serotype carriage was based on culture or Quellung-reaction results.

^c^ The number of samples was used for the analyses regarding Sp-carriage predictors; the number of isolates was used for the analyses regarding PCV13-non-PCV10-VT-carriage predictors.

^d^ Confidence intervals that do not overlap the null value of OR = 1 are indicated in bold.

^e^ Information on common cold symptoms was not available for five children.

^f^ Based on culture-results or Quellung-reaction.

^g^ Based on the combination of culture and PCR-results.

^h^ Information on siblings was not available for 40 children.

^i^ Child with a history of AOM.

^j^ Information on history of AOM was not available for 78 children.

^k^ Use of antibiotics in the 3 months before sampling.

^l^ Information on use of antibiotics in the 3 months before sampling was not available for 157 children.

^m^ Information on vaccination status was missing for one child.

Besides the shown confounders, other variables were assessed, but not significant in univariate analysis: preterm delivery, previous hospitalisation, age-appropriate vaccination, carriage of *Streptococcus pyogenes *(based on culture-results or Quellung-reaction), parental smoking, breastfeeding.

### Trends over time in carriage prevalence of *Streptococcus pneumoniae* serotypes

Among the Sp-carriers based on Quellung-results, a decreasing trend (p < 0.001) in the proportion of non-PCV13-VTs (94.6%, 438/463 isolates in 2016 vs 96.9%, 594/613 isolates in 2016–2017 vs 89.7%, 599/668 isolates in 2017–2018) was accompanied by an increasing trend (p < 0.001) in the proportion of PCV13-non-PCV10-VTs (3 + 6A + 19A: 0.9%, 4/463 isolates in 2016 vs 1.6%, 10/613 isolates in 2016–2017 vs 7.8%, 52/668 isolates in 2017–2018; Figure 1). 

**Figure 3:**
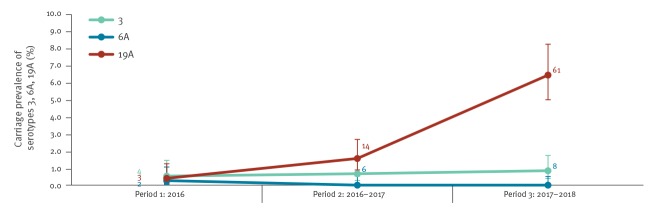
Carriage prevalence of PCV13-non-PCV10 vaccine serotypes 3, 6A, 19A^a^, Belgium, 2016–2018 (n = 761 in 2016, 904 in 2016–2017, 956 in 2017–2018)

^a^ Carriage prevalence was determined from the results of Quellung-reaction and PCR combined.

Among the latter, serotype 19A was most frequently identified (87.9%, 58/66 isolates), followed by serotype 3 (10.6%, 7/66 isolates), and serotype 6A, which was identified once (1.5%, 1/66 isolates). The Quellung-reaction and PCR combined results of these serotypes among DCC-children’s isolates (Figure 3) showed a stable carriage prevalence for serotypes 3 (0.5%, 4/761 isolates in 2016 vs 0.7%, 6/904 isolates in 2016–2017 vs 0.8%, 8/956 isolates in 2017–2018) and 6A (0.3%, 2/761 isolates; 0.0%; 0.0%), but a significant increase (p < 0.001) was observed for serotype 19A (0.4%, 3/761 isolates in 2016 vs 1.5%, 14/904 isolates in 2016–2017 vs 6.4%, 61/956 isolates in 2017–2018).

Error bars indicate 95% confidence intervals, numbers added to the respective line charts indicate the total number of isolates for the respective serotype during the indicated study period.

Based on Quellung-results, non-PCV13-VTs dominated Sp-carriage over the entire study period. The serotypes 23B, 23A, 11A, 15B, 15A, and 10A constituted nearly 50% of the total non-PCV13-VTs among Sp-carriers in all three periods. The separate proportions of the different serotypes identified among Sp-carriers fluctuated over the study period except for three serotypes. The proportions of serotypes 19A and 6C consistently increased (p < 0.001); from 0.4% (2/463 isolates) in 2016, to 1.5% (9/613 isolates) in 2016–2017, to 7.0% (47/668 isolates) in 2017–2018 for serotype 19A and from 0.9% (4/463 isolates) in 2016, to 1.5% (9/613 isolates) in 2016–2017, to 5.8% (39/668 isolates) in 2017–2018 for serotype 6C. The proportion of serotype 15A consistently decreased (p = 0.042); from 6.7% (31/463 isolates) in 2016 to 5.2% (32/613 isolates) in 2016–2017 to 3.4% (23/668 isolates) in 2017–2018.

### *Streptococcus pneumoniae* and its antimicrobial non-susceptibility

**Figure 4:**
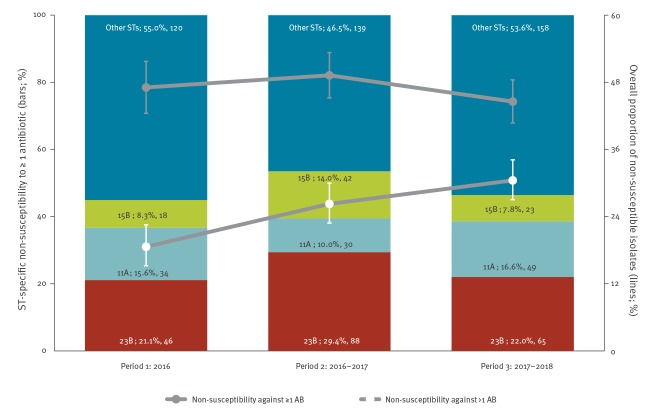
Non-susceptibility against at least one (n = 463 isolates in 2016, 607 in 2016–2017, 662 in 2017–2018) or more than one (n = 462 isolates in 2016, 609 in 2016–2017, 666 in 2017–2018) of the tested antibiotics^a^ and the dominating serotypes among strains of *Streptococcus pneumoniae* non-susceptible to at least one antibiotic^b^, Belgium, 2016–2018

The proportion of Sp-strains that were non-susceptible against any of the five tested antibiotics remained stable over the study period (47.1%, 218/463 isolates in 2016 vs 49.3%, 299/607 isolates in 2016–2017 vs 44.6%, 295/662 isolates in 2017–2018), whereas non-susceptibility against more than one antibiotic increased (18.6%, 86/462 isolates in 2016 vs 26.3%, 160/609 isolates in 2016–2017, vs 30.5%, 203/666 isolates in 2017–2018; p < 0.001). Non-susceptibility against levofloxacin (cut-off MIC > 2.00 mg/L) was inexistent. Non-susceptibility against penicillin (cut-off MIC > 0.06 mg/L; 13.4%, 62/463 isolates in 2016 vs 19.2%, 117/609 isolates in 2016–2017 vs 18.5%, 123/666 isolates in 2017–2018; p = 0.041), erythromycin (17.3%, 80/463 isolates in 2016 vs 16.1%, 98/608 isolates in 2016–2017 vs 22.0%, 146/664 isolates in 2017–2018; p = 0.028), and tetracycline (11.7%, 54/463 isolates in 2016 vs 12.6%, 77/610 isolates in 2016–2017 vs 20.0%, 133/666 isolates in 2017–2018; p < 0.001) increased over the study period, whereas non-susceptibility against trimethoprim/sulfamethoxazole fluctuated over the study period (35.2%, 163/463 isolates in 2016 vs 40.3%, 246/610 isolates in 2016–2017 vs 30.3%, 202/666 isolates in 2017–2018; p = 0.042). Nevertheless, trimethoprim/sulfamethoxazole was the antibiotic against which most strains (35.1%; 611/1,739) were non-susceptible over the entire study period. The serotypes (based on Quellung-reaction) that were most often found to be non-susceptible against at least one of the tested antibiotics among Sp-carriers are shown in Figure 4.

AB: antibiotic; ST(s): serotype(s).

^a ^Non susceptibility to antibiotics was assessed using cultures and the overall proportions of non-susceptible isolates (against ≥ 1 and > 1 antibiotic) are represented by lines.

^b ^Dominating serotypes among strains of *Streptococcus pneumoniae* non-susceptible to ≥ 1 antibiotic as well as all the other remaining serotypes are shown within the chart bars. For each particular serotype presented, the serotype number, followed by the proportion and number of isolates for this serotype is indicated. Serotypes were determined by Quellung-reaction.

Error bars indicate 95% confidence intervals. A minimum inhibitory concentration (MIC) of > 0.06 mg/L (penicillin) and > 2.00 mg/L (levofloxacin) was interpreted as non-susceptible.

Over the 3-year study period, 23.2% (13/56 19A-strains) of the 19A-strains were non-susceptible to more than one of the tested antibiotics, whereas two 19A-strains (3.6%; 2/56 19A-strains) were non-susceptible to only one of the tested antibiotics; erythromycin and trimethoprim/sulfamethoxazole for the respective strains. The isolated 19A-strains were most frequently non-susceptible to erythromycin (93.3%; 14/15 19A-strains), followed by tetracycline (86.7%; 13/15 19A-strains), trimethoprim/sulfamethoxazole (46.7%; 7/15 19A-strains), and penicillin (20.0%; 3/15 19A-strains).

## Discussion

In this pneumococcal carriage study, we evaluated during 3 years any changes in the nasopharyngeal carriage prevalence, serotype distribution and antimicrobial (non-)susceptibility of Sp in healthy children (aged 6–30 months) attending DCCs in Belgium, from 2016 onwards, i.e. during and immediately after a PCV13-to-PCV10 vaccination programme switch. Common co-colonising bacteria were followed as well, with a special focus on Hi.

Demographics and clinical characteristics of the study population showed higher percentages of common cold symptoms in 2016–2017 and 2017–2018 compared with 2016, which might be due to differences in sampling period. In 2016, nasopharyngeal samples were taken during spring (March–July), whereas the subsequent periods encompassed autumn and winter (November–March), during which common cold frequently occurs. The higher percentages of AOM-history in 2016 compared with the other periods might be due to recruitment of (on average) older children in this year compared with the subsequent periods and in 2016 more AB-treatments in the 3 months before sampling were observed. With regard to breastfeeding, it is unclear why it is more frequent in 2017–2018 compared with the previous periods. It is likely that several factors contributed to fluctuations in breastfeeding practices, one of which may be the coinciding extensive campaigns (personal communication: Marc Hainaut, 24 Oct 2018) on the importance of breastfeeding in Brussels’ maternity hospitals (in 2017–2018, > 54% of the included Brussels children were breastfed for more than 6 months).

During the study period, the vaccination status of the child populations gradually changed; from mainly PCV13-vaccinated children in 2016 to mainly PCV10-vaccinated children in 2017–2018. Sp-carriage prevalence was consistently high (> 75%) over the study period. Real-life carriage in DCC might be slightly lower since children who were treated with oral ABs in the 7 days before sampling could not take part in the study.

In contrast, the carriage prevalence of Hi increased significantly over the study period (especially in the two oldest age categories), despite the increasing number of children vaccinated with PCV10, containing the NTHi protein D. Other reports also indicated the absence of PCV10-induced protection against NTHi [39-41].

The predictors of Sp-carriage identified in our study include study period, sex, siblings, common cold symptoms, use of antibiotics, Hi-carriage, Mc-carriage, Sa-carriage and confirm the findings of other reports (besides study period and sex) [5,19,20,42,43].

Based on Quellung-reaction, the proportion of non-PCV13-VTs decreased significantly over the study period, associated with an increase in the proportion of the three PCV13-non-PCV10-VTs 3, 6A, 19A (mainly 19A). We verified for confounders (preterm delivery, previous hospitalisation, age-appropriate vaccination, GAS-carriage, parental smoking, breastfeeding, and the variables shown in Table 2) that could have caused increased PCV13-non-PCV10-VT-carriage, but could not identify any besides study period, which strengthens the hypothesis that the increase was caused by the vaccine switch.

Serotype 19A became the most frequent vaccine serotype in 2017–2018 and its PCR-based prevalence rose from 0.4% in 2016 to 6.4% in 2017–2018. According to surveillance data on IPD from the NRC in 2017, serotype 19A was the second most frequent serotype (after serotype 12F) among IPD-isolates of children younger than 2 years of age. While no increase in serotype 19A frequency had been noted since the introduction of PCV13 in 2011, an increase was observed for the first time in 2016, when the frequency changed from 2.1% in 2016 to 14.2% in 2017 [44]. These findings were confirmed in 2018, 2 to 3 years after the switch from PCV13 to PCV10 [45]. In addition to being reported as an invasive serotype, 19A has also been reported as a serotype that is frequently non-susceptible to antimicrobials [46]. Nevertheless, in our study other serotypes dominated among the non-susceptible strains (23B, 11A, 15B) as they were more prevalent than 19A. Since we excluded children who were treated with oral ABs in the 7 days before sampling, we possibly missed some non-susceptible strains (including 19A). Besides for serotype 19A, a consistently increasing proportion was also observed for the carriage of serotype 6C, which increased from 0.9% in 2016 to 5.8% in 2017–2018. Sweden, where PCV13 is used in some regions, while PCV10 is used in others, also reported an increase in serotype 6C in PCV10-regions [47]. This could have implications for the non-vaccinated elderly, as reported in Finland, where serotypes 19A and 6C are frequently isolated from IPD in adults older than 65 years [48].

Our results should be interpreted in the context of several limitations. First, a decreasing age trend was introduced by recruiting older children in 2016 in order to include a maximum of children vaccinated with PCV13 and for the same reason, Wallonia was over-recruited in 2016. Since this is intrinsic to our study design, we cannot adjust for this: the sampling probabilities did not allow re-weighting for any of the analyses because no children were sampled in the youngest age category in Flanders and in Brussels in 2016.

Furthermore, the Sp-carriage prevalence was stable within the different age categories (6–12, 13–24, 25–30 months) over the study period, but the Hi-carriage prevalence in the two oldest age categories increased over the study period. Second, we over-recruited in Wallonia in 2016 to enlarge the PCV13-vaccinated population, but in the first season no regional differences in overall Hi-carriage or pneumococcal carriage, and vaccine type carriage were found. Third, a comparative analysis based on the children’s vaccination schedule was not performed due to the small size of these subpopulations in either 2016 (few PCV10-vaccinated children) or 2017–2018 (few PCV13-vaccinated children). Finally, a 3-year study period is short to completely exclude natural fluctuations in the carriage prevalence of specific serotypes; a follow-up study should confirm whether or not a new equilibrium is reached.

Despite these limitations, our study allowed to monitor the impact of the PCV13-to-PCV10 vaccine switch on nasopharyngeal carriage, serotype distribution, and antimicrobial (non-)susceptibility of Sp. This is a complementary activity to the analysis of IPD-surveillance data, thus providing further basis for policy making on pneumococcal vaccination programme options.

## Conclusions

As the proportion of children vaccinated exclusively with PCV10 increased, the proportion of the serotypes not included in PCV13 decreased over the 3-year study period, mainly due to an increase in the carriage prevalence of serotype 19A.

## NPcarriage Study Group

David Tuerlinckx, CHU Dinant-Godinne, Université Catholique de Louvain, Yvoir, Belgium; Adam Finn, University of Bristol, School of Cellular and Molecular Medicine, Bristol, UK; Koen Van Herck, Department of Public Health and Primary Care, Ghent University, Gent, Belgium, Centre for the Evaluation of Vaccination, Vaccine and Infectious Disease Institute, University of Antwerp, Wilrijk, Belgium; Robert Cohen, Université Paris Est, IMRB-GRC GEMINI, 94000 Créteil, France; Christine Lammens, Laboratory of Medical Microbiology, Vaccine and Infectious Disease Institute, University of Antwerp, Wilrijk, Belgium.
